# The use of Versius CMR for pelvic surgery: a multicentric analysis of surgical setup and early outcomes

**DOI:** 10.1007/s00345-023-04730-3

**Published:** 2024-01-13

**Authors:** Maria Chiara Sighinolfi, Maurizio De Maria, Iacopo Meneghetti, Mauro Felline, Andrea Pisani Ceretti, Luca Mosillo, Chiara Catalano, Alessandro Morandi, Tommaso Calcagnile, Enrico Panio, Mattia Sangalli, Filippo Turri, Stefano Terzoni, Simone Assumma, Luca Sarchi, Margarita Afonina, Annamaria Marconi, Paolo Pietro Bianchi, Salvatore Micali, Bernardo Rocco, Giorgia Gaia

**Affiliations:** 1 Unit of Urology, ASST Santi Paolo and Carlo, Milan, Italy; 2 Unit of Urology, Apuane Hospital, Massa, Italy; 3 Unit of Gynecology, ASST Santi Paolo and Carlo, Milan, Italy; 4 Unit of General Surgery, ASST Santi Paolo and Carlo, Milan, Italy; 5 Unit of Urology, Azienda Ospedaliero Universitaria di Modena, Modena, Italy

**Keywords:** Pelvic surgery, Versius CMR, New robotic systems

## Abstract

**Introduction:**

Versius CMR is a novel robotic system characterized by an open surgical console and independent bedside units. The system has potentials of flexibility and versatility, and has been used in urological, gynecological, and general surgical procedure. The aim is to depict a comprehensive analysis of the Versius system for pelvic surgery.

**Methods:**

This is a study involving two Institutions, ASST Santi Paolo and Carlo, Milan, and Apuane Hospital, Massa, Italy. All interventions performed in the pelvic area with the Versius were included. Data about indications, intra-, and post-operative course were prospectively collected and analyzed.

**Results:**

A total of 171 interventions were performed with the Versius. Forty-two of them involved pelvic procedures. Twenty-two had an oncological indication (localized prostate cancer), the remaining had a non-oncological or functional purpose. The mostly performed pelvic procedure was radical prostatectomy (22) followed by annexectomy (9). No intra-operative complication nor conversion to other approaches occurred. A Clavien II complication and one Clavien IIIb were reported. Malfunctioning/alarms requiring a power cycle of the system occurred in 2 different cases. An adjustment in trocar placement according to patients’ height was required in 2 patients undergoing prostatectomy, in which the trocar was moved caudally. In two cases, a pelvic prolapse was repaired concomitant with other gynecological procedures.

**Conclusions:**

Pelvic surgery with the Versius is feasible without major complications; either dissection and reconstructive steps could be accomplished, provided a proper OR setup and trocar placement are pursued. Versius can be easily adopted by surgeons of different disciplines and backgrounds; a further multi-specialty implementation is presumed and long-term oncological and functional outcomes are awaited.

## Introduction

Robotic surgery has revolutionized the treatment of several diseases given the improved clinical outcomes, such as less blood loss, shorter length of hospital stay and fewer complications compared with laparoscopy or open approach. The main limitation has been the high cost due to purchase and maintenance of the da Vinci™ robotic surgical system (Intuitive Surgical, Sunnyvale, CA, USA) that represented the only alternative of the global market so far. After the Intuitive’s patent expiry, novel robotic platforms became available, including the Revo-I, the Senhance, the Versius, Avatera, Hinotori, and Hugo RAS. Some of these new systems share features differing from those of the da Vinci, both in terms of console, robotic units, and arm; thus, the introduction of novel platforms poses specific challenges to be addressed by the whole operating team; information and training about indications, setup, and outcomes are mandatory.

The Versius CMR is one of those new systems that has been approved in the UK in 2018. It displays an open surgical console with hand controllers and a head-up display (HUD). Versius is a multi-modular system with independent bedside unit (BSU), one dedicated to visualization with an endoscopic camera. The HUD provides the surgeon with a three-dimensional, high-definition visualization. The system carries the potentials of versatility, due to the open console design—improving communication in the OR—and adaptability with up to 4 BSU located around the operating table with multiple allowed configurations. The hand controllers are ergonomically designed and, unlike the Da Vinci and other new systems, accommodate all functions—including camera movement and energy delivery—without pedal control [1–3].

After strong evidence of feasibility developed in the pre-clinical setting [4], clinical outcomes from the official “Versius Robotic Surgical System Registry” are yet to come; a low rate of conversion to alternative technique, serious adverse events, and 90-day mortality is anticipated [5].

However, even if recognized viable from wide series, the description of each individual Versius procedures with specific challenges is still crucial.

The aim of the article is to report a comprehensive analysis of the Versius system for pelvic surgery, by describing indications, setup, and early outcomes in a multicentric study.

## Methods

This is a retrospective observational study involving two Institutions, ASST Santi Paolo and Carlo, Milan, and at the Apuane Hospital, Massa, Italy.

The Versius CMR was installed by September, 2022, at the former institution, and by 2021 in the latter. The console surgeons involved were urologists (BR and DM), gynecologists (GG, MF), and general surgeons (AP). All except two were prior Da Vinci users; the latter (AP an MF) had an extensive laparoscopic background.

All interventions performed in the pelvic area with the Versius were included in the analysis. Data about indications, intra-, and post-operative course were prospectively collected.

### Training

The training involved the whole OR Team (console surgeon, bedside assistant, scrub nurse, circulating nurse). For surgeons, the Versius training package consisted of a 3.5-day program following 10 h of online didactic training; it includes dry box exercises and wet lab sessions simulated in an operating room using cadaveric models.

### Interventions

Urologic pelvic procedures, gynecological procedures, pelvic procedures from general surgeons were considered. Colectomy and hernia repair were excluded. Interventions could be either performed for oncological or non-oncological purposes; reconstructive interventions with the use of devices (i.e., mesh) were included as well.

### Data collection and analysis

The following variables were collected:

- Demographics: age, gender;
- Surgical indications
- Intra-operative data: OR setup, number of ports, number of robotic arms, console time, estimated blood loss (EBL), conversion to alternative approaches, complications invoking a change in surgical strategy; use of additional devices for repair (i.e., mesh); malfunctioning of the system requiring a re-start
- Post-operative data: complications classified by Clavien– Dindo, transfusion rate, length of stay (LOS), 30- and 90-day re-admission

Data were inserted in a dedicated data base (Excel, Microsoft) and analyzed by an external reviewer (ST) with formal habilitation as a coordinator with the Versius and the Da Vinci systems. A descriptive analysis of all variables was performed. Console Time, EBL, and LOS were provided as mean value and range.

## Results

**Fig. 1:**
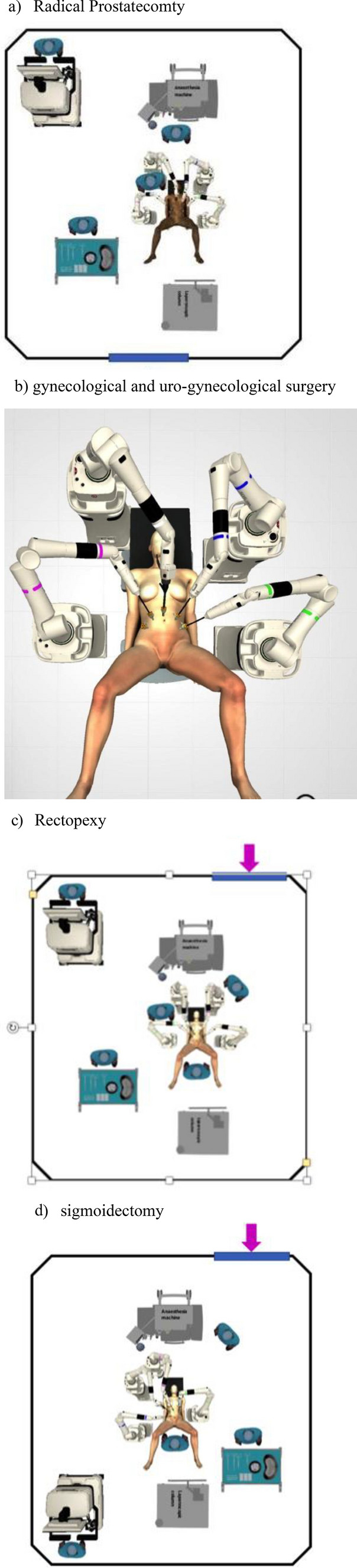
OR setup for prostatectomy (**a**), gynecologic surgery (**b**), rectopexy (**c**), signoidectomy (**d**)

Overall, a total of 171 interventions were performed with the Versius CMR at the ASST Santi Paolo and Carlo (120) and at the Apuane Hospital (51). Forty-two of them involved pelvic procedures. A full list of interventions stratified by center and specialty is reported in Table 1. Twenty-two interventions had an oncological indication (localized prostate cancer) whereas the remaining ones had a non-oncological or functional purpose. The mostly performed procedures were radical prostatectomy (22) and annexectomy (9). The OR setup for urological, gynecological and general surgical procedure (rectopexy and sigmoidectomy) is depicted in Fig. 1a–d, respectively. No intra-operative complication invoking a change in surgical strategy occurred nor conversion to open or laparoscopic surgery. Two Clavien II and one IIIb complications were evident (pelvic hematoma requiring transfusion, a urinary tract infection treated with antibiotic therapy and a bowel obstruction due to port-site herniation). Table 2 reports console time, EBL, complications, and LOS stratified by procedure. Malfunctioning/alarms requiring the whole system re-activation occurred in 2 different cases. An adjustment in trocar placement according to patients’ height was required in 2 patients undergoing RALP, in which the trocar was moved caudally. In two cases, a pelvic organ prolapse (POP) was repaired concomitant with other gynecological procedures: an hysterectomy, bilateral salpingo-oophorectomy, and lateral suspension with a titanium-coated polypropylene mesh, according to Dubuisson technique. A single case, a 53-year-old woman, underwent a ventral rectopexy for full-thickness rectal external prolapse.

**Table 1:** Procedures stratified by Center

Procedure	Number	Center	Details
Radical prostatectomy	22	ASST Santi Paolo and Carlo (4) Apuane Hospital (18)	
Annexectomy	9	ASST Santi Paolo and Carlo	Bilateral in 8 cases
Surgery for Endometriosis	2	ASST Santi Paolo and Carlo	
Hysterectomy	3	ASST Santi Paolo and Carlo	Concomitant POP repair in 1 case
Ovarian cyst enucleation	4	ASST Santi Paolo and Carlo	Bilateral in 2 cases
Dubuisson repair	1	ASST Santi Paolo and Carlo	
Ventral rectopexy	1	ASST Santi Paolo and Carlo	
Sigmoidectomy	1	ASST Santi Paolo and Carlo

**Table 2:** Peri-operative outcomes stratified by procedure

Procedure	N° robotic arms	Console time	EBL	Complications	LOS
Radical prostatectomy	4	201 (130–242)	140 (100–550)	1 pelvic hematoma 1 UTI 1 port-site herniation	4 (3.75–7)
Annexectomy	4	58 (31–77)	0 (0–0)	No	2
Hysterectomy	4	142 (122–176)	133 (50–200)	No	2
Ovarian cyst enucleation	3 in 2 cases, 4 in 1	115 (76–147)	37 (0–62)	No	2
Surgery for Endometriosis	4	217 (210–225)	50 (0–100)	No	2
Dubuisson Lateral suspension	4	170	0	No	3
Ventral rectopexy	4	94	100	No	3
Sigmoidectomy	4	80	100	No	5

Positive margin (PM) rate can be evaluated for radical prostatectomies. The outcomes are variable, one series showed 83.3% of PM rate on 18 cases (11 pT3, published data) [6] and in the other one (4 pT2c cases), no PM were found. At a follow-up ranging from 9 to 11 months, a single case of biochemical recurrence was noticed. One re-admission at 30 days (herniation) was recorded, and none at 90 days.

## Discussion

The current series represents the first one completely focused on pelvic surgery with the new Versius surgical system.

Sparse reports evaluating single indications with Versius [7–9] are available yet; similarly, some series reported on the use of Versius for abdominal surgery, the majority including preliminary procedures—hernia repair, cholecystectomy—to become familiar with the system [10, 11]. In the present series, we intentionally addressed the pelvic area, to evaluate if robotic surgery with a novel platform may address the challenges posed by such interventions, often made up by complex tasks (dissection and reconstruction) in small spaces with limited accessibility and range of movements. A comprehensive analysis of pelvic approach with Versius has been already reported in the pre-clinical setting: Vasdess et al. [11] described the possible setup for prostate surgery on cadaveric models, exploring multiple options of trocar placement with either 3- and 4-arm configurations. To note, authors evaluated also the feasibility of radical cystectomy (even if in vivo cystectomy has not yet performed so far with the Versius).

Our series confirms the feasibility of Versius pelvic surgery in different settings without major complication; actually, we did not face the need for conversion to other approaches. In preliminary series, conversion has been reported in up to 4–6% of Versius cases [12, 13] together with a certain variable degree of adverse events [13–15]. Herein, a single Clavien IIIb complication was reported (bowel herniation through abdominal wall requiring surgical repair); the occurrence is seemingly unrelated to the use of a novel robotic system. As far as surgical margin status is concerned, radical prostatectomy is the only oncological intervention we included. In this setting, PM rate from a single institution series appears of importance (83.3%); the small sample size and a high rate of pT3 could have accounted for the occurrence [6]. Moreover, PM is counterbalanced by low BCR, (a single case showing a raise in post-operative PSA out of 22 RALP), provided long-term PSA is still awaited.

Unlike other series dealing with the Versius, some complex cases have been performed as well in the present article: this is the case of surgery for endometriosis and of a case of post-radiation sigmoidectomy.

Some specifics to pelvic surgery may apply to the Versius system.

Surgical instruments are shorter than those of the Da Vinci (30 cm): the issue should be considered and an estimation of the distance to the target area (and to other areas to be reached, i.e., pelvic nodal dissection) should be pre-planned. Port placement can be, therefore, moved caudally than the conventional Da Vinci configuration, especially considering patient’s height. In two RALP cases, we had to move one port more caudally to make the instrument reach the target area (urethral stump). The BMI of the patient is another issue that needs to be taken into account: in the current series, extreme BMIs have been successfully managed with a prior accurate planning of port and BSU placement. This was the case of gynecological surgery, which encompassed a range of BMI from 16 to 43 (non-published data).

One of the major concerns of multi-modular robotic system is the likelihood of external clashing between arms, a point somehow counterbalancing the versatility invoked by new systems with independent units [16]. Overall, whereas the Da Vinci Xi allows for a linear trocar placement with a standardized docking, such a step with the Versius should be accurately planned to minimize clashing or limitation in instruments motion [16]. Another feature typical of the Versius system is the docking of the robotic arm to the instrument and not to the trocar, as occurs with the Da Vinci or Hugo RAS; if the use of a non-dedicated trocar may represent an advantage, on the other side collisions between the robotic arm and the skin of the patient are recognized by the system and given as an alarm. The issue should be taken into account as well during the setup, especially in pelvic surgery in which a Trendelenburg of the patient is required and angles with the trocar could be relevant.

Beyond technical details, some general considerations may arise from the current experience.

Versius can be used by surgeons from different disciplines and it heavily comprises diverse surgical specialties; the same occurs with the Da Vinci, even if major users have initially been urologists [1]. It is representative that in a center owning three robotic systems such as the ASST Santi Paolo and Carlo (Da Vinci, Versius CMR and Hugo RAS), the majority of Versius cases have been accomplished by gynecologists and general surgeons.

In line with these findings, laparoscopists seem to be those surgeons mostly advantaging from the introduction of Versius. The instruments are designed to mimic the articulation of the human arm and the wristed joints with seven degrees of freedom overcome the difficulties of laparoscopic surgery. The use of laparoscopic trocars—not robotic dedicated—raises opportunity for a hybrid procedure; thus, laparoscopists appreciate Versius features designed to simplify laparoscopic surgery.

Severe malfunctioning of the Versius has been reported in few cases and has been fixed with the re-start of the system (2 cases). If compared to the Da Vinci, it should be recognized that the latter has reached its fourth generation, whereas the Versius system is at its very first one; technological improvement and optimization are awaited. Noticeably, similar considerations may apply also to other novel robotic platforms that entered the market within the last two years.

As far as the Versius is concerned, technological updates are already developing: an improvement in arm clash recovery has been recently released—i.e., maintenance of arm engagement from the surgeon in case of clash—and other enhancements are yet to come, such as improvement of bipolar devices and the implementation of an energy sealer device [12].

The article is not devoid of limitations.

First, the small sample size and short follow-up preclude any conclusion about mid- and long-term outcomes. The different kind of surgeries included make outcome assumptions weak; however, the report of long-term clinical follow-up was beyond the purpose of the paper, that merely aimed to address the feasibility of Versius surgery in the pelvic area.

Second, no matched comparison with the Da Vinci nor a subjective comparison is herein provided. Some console surgeons (BR, DM, GG) and assistants (MCS, FT, MS) are currently operating on multiple platforms, but a subjective perception about systems is not herein provided. However, an objective comparison of the first RALP cases with the Versius and Hugo RAS has been described by our group throughout the metrics developed by the ERUS working group for radical prostatectomy [17]. Further comparative analysis of robotic systems is expected in the very next future, to highlight differences and peculiarity of each platform trying to draw definite outcomes.

## Conclusions

From the present series, pelvic surgery with the Versius system is feasible without severe intra- or peri-operative complications; long-term oncological and functional outcomes are yet to be defined. All steps of pelvic surgery are reproducible with the Versius, provided a proper surgical setup and trocar placement are pursued. Versius can be easily adopted by surgeons of different disciplines and background within the same institution; a further multi-specialty implementation is expected, and a cost analysis is awaited to highlight its future role into healthcare systems.

## Data Availability

Data will be shared upon appropriate request.
