# Correction: The use of Versius CMR for pelvic surgery: a multicentric analysis of surgical setup and early outcomes

**DOI:** 10.1007/s00345-024-04850-4

**Published:** 2024-02-19

**Authors:** Maria Chiara Sighinolfi, Maurizio De Maria, Iacopo Meneghetti, Mauro Felline, Andrea Pisani Ceretti, Luca Mosillo, Chiara Catalano, Alessandro Morandi, Tommaso Calcagnile, Enrico Panio, Mattia Sangalli, Filippo Turri, Stefano Terzoni, Simone Assumma, Luca Sarchi, Margarita Afonina, Annamaria Marconi, Paolo Pietro Bianchi, Salvatore Micali, Bernardo Rocco, Giorgia Gaia

**Affiliations:** 1 Unit of Urology, ASST Santi Paolo and Carlo, Milan, Italy; 2 Unit of Urology, Apuane Hospital, Massa, Italy; 3 Unit of Gynecology, ASST Santi Paolo and Carlo, Milan, Italy; 4 Unit of General Surgery, ASST Santi Paolo and Carlo, Milan, Italy; 5 Unit of Urology, Azienda Ospedaliero Universitaria di Modena, Modena, Italy

**Correction: World Journal of Urology (2024) 42:31** 10.1007/s00345-023-04730-3

In this article, the author name Iacopo Meneghetti was incorrectly published as Jacopo Meneghetti.

Now, the original article has been corrected.

